# Development of the follicular basement membrane during human gametogenesis and early folliculogenesis

**DOI:** 10.1186/s12861-015-0054-0

**Published:** 2015-01-21

**Authors:** A Marijne Heeren, Liesbeth van Iperen, Daniëlle B Klootwijk, Ana de Melo Bernardo, Matthias S Roost, Maria M Gomes Fernandes, Leonie A Louwe, Carina G Hilders, Frans M Helmerhorst, Lucette A J van der Westerlaken, Susana M Chuva de Sousa Lopes

**Affiliations:** Department of Anatomy and Embryology, Leiden University Medical Center, Einthovenweg 20, 2333 Leiden, ZC The Netherlands; Department of Gynaecology, Leiden University Medical Center, Albinusdreef 2, 2300 Leiden, RC The Netherlands; Current address: Department of Obstetrics and Gynaecology, VU University Medical Center, De Boelelaan 1118, 1081 Amsterdam, HZ The Netherlands; Department of Gynaecology, Reinier de Graaf Hospital, Reinier de Graaf 3-11, 2625 Delft, AD The Netherlands; Department for Reproductive Medicine, Ghent University Hospital, De Pintelaan 185, 9000 Ghent, Belgium

**Keywords:** Human, Germ cells, Adult ovary, Follicles, Extracellular matrix, Gonads

## Abstract

**Background:**

In society, there is a clear need to improve the success rate of techniques to restore fertility. Therefore a deeper knowledge of the dynamics of the complex molecular environment that regulates human gametogenesis and (early) folliculogenesis *in vivo* is necessary. Here, we have studied these processes focusing on the formation of the follicular basement membrane (BM) *in vivo*.

**Results:**

The distribution of the main components of the extracellular matrix (ECM) collagen IV, laminin and fibronectin by week 10 of gestation (W10) in the ovarian cortex revealed the existence of ovarian cords and of a distinct mesenchymal compartment, resembling the organization in the male gonads. By W17, the first primordial follicles were assembled individually in that (cortical) mesenchymal compartment and were already encapsulated by a BM of collagen IV and laminin, but not fibronectin. In adults, in the primary and secondary follicles, collagen IV, laminin and to a lesser extent fibronectin were prominent in the follicular BM.

**Conclusions:**

The ECM-molecular niche compartimentalizes the female gonads from the time of germ cell colonization until adulthood. This knowledge may contribute to improve methods to recreate the environment needed for successful folliculogenesis *in vitro* and that would benefit a large number of infertility patients.

**Electronic supplementary material:**

The online version of this article (doi:10.1186/s12861-015-0054-0) contains supplementary material, which is available to authorized users.

## Background

During human embryonic development, the somatic cellular component of the gonads develops from the gonadal primordia, whereas the primordial germ cells (PGCs) originate outside the gonadal primordia. The PGCs present in the posterior part of the yolk sac, close to the allantois and hindgut wall, need to migrate to colonize the prospective ovary or testis. Interestingly, when the PGCs arrive in the gonadal primordia, male and female gonads are morphologically identical, but due to extensive remodelling by week 12 of gestation, W12 (i.e. 10 week of fetal development), the gonads become clearly dimorphic. In females, the number of germ cells increases until W20, but declines from W20 until birth [1,2]. However, every woman is born with still about a million of resting primordial follicles in their ovaries [3] and it is only in cycling females (the menstrual cycle starting in average at the age of 12 years) that the follicles sequentially mature, leading to the ovulation every month (each menstrual cycle) of about one mature oocyte. *In vitro*, gametogenesis has proven very challenging to mimic and knowledge to improve each step of the process in particular concerning human gametogenesis is needed.

During gonadogenesis and gametogenesis, the formation of both a defined somatic compartment and a defined germ cell compartment is paramount for the dual functionality of the gonad, the production of mature gametes and its role as endocrine gland in the hypothalamic-pituitary-gonadal axis. In the testis, the germ cell compartment formed by a group of seminiferous tubes, is surrounded by a basement membrane (BM) of extracellular matrix (ECM), whose major components are laminin, collagen IV and fibronectin, that provide structural support [4-6]. In the ovary, the follicular BM also provides structural support to the developing follicles, maintaining cellular organization, but it also regulates the highly selective process of follicular growth and maturation, because it binds and retains growth factors and hormones that affect the development of both the granulosa cells and the oocyte itself [2,7-9].

The continual remodelling of the ECM in the follicular BM during folliculogenesis up to ovulation has been studied in various animal models, but in humans, most of the studies on the distribution and composition of the ECM in the ovary have examined pre-ovulatory follicles, corpora lutea and the surrounding ovarian stroma [10-14] and knowledge about the dynamics and composition of the BM during gametogenesis and early folliculogenesis is lacking.

Here, we have focused on the development of the human follicular BM and its associated ECM main components during first trimester, second trimester and early folliculogenesis by immunofluorescence. Our results reveal a higher degree of similarity regarding the initial compartmentalization of human gonads in ovarian cords and seminiferous cords. Here, we underscore the existence of a follicular BM from the time that the first primordial follicles emerge in the ovarian (cortical) mesenchyme. Understanding the dynamics in follicular BM may prove important to develop successful protocols to recreate the environment needed for successful folliculogenesis *in vitro* [15].

## Results

### Collagen IV-rich basement membranes showed the presence of ovarian cords in fetal female gonads at W9-W12

**Figure 1:**
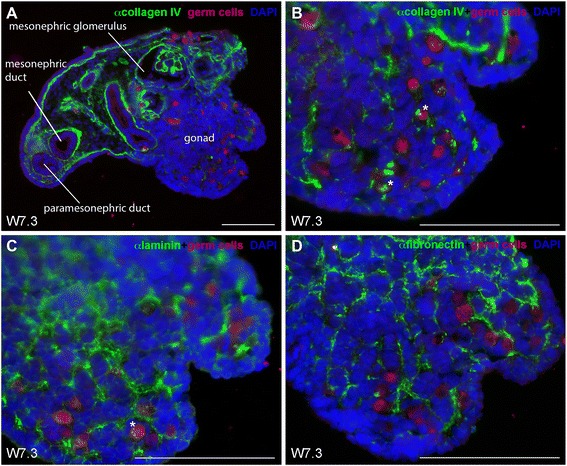
**Collagen IV, laminin and fibronectin expression in human fetal female gonads during early development W7.3. (A,B)** Collagen IV (green) expression identified the mesonepheric glomerulus, mesonephric and paramesonepheric ducts, and is present in the gonad. **(C,D)** laminin (green) **(C)**, and fibronectin (green) **(D)** expression during early development of female gonads (W7.3). Germ cells (red) were identified by the early marker OCT4 (nuclear) and late marker VASA (cytoplasmic). The same secondary antibody was used to detect OCT4 and VASA, because the two proteins localize to different cellular compartments (nucleus and cytoplasm, respectively) and their expression can therefore be clearly distinguished. The white asterisks marked individual germ cells expressing collagen IV and laminin. Scalebars are 100 μm.

At W6-7, we observed a low number of germ cells colonizing the human female gonads (Figure 1). Many structures in the mesonephros, including the mesonephric glomeruli, tubuli and duct, were clearly delineated by a BM rich in collagen IV, however the developing gonad contained only few collagen IV fibrils without an apparent organization (Figure 1A and 1B). Laminin and fibronectin also seemed ubiquitously present in the female gonad (Figure 1C and 1D).

**Figure 2:**
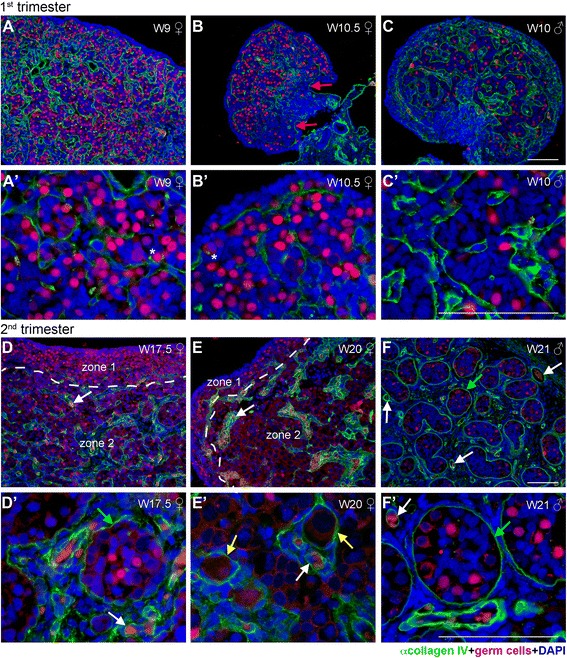
**Collagen IV expression in human fetal female and male gonads during first and second trimester. (A-C)** Longitudinal sight of W9 **(A,A’)** and transversal sight of W10.5 **(B,B’)** female gonad showed a distinctive expression pattern of collagen IV (green). **(C,C’)** Collagen IV (green) detected in the BM surrounding the developing seminiferous tubes in male gonad of first trimester (W10). **(D-F)** Collagen IV (green) expression in the BM of ovarian cords in female gonads **(D,D’,E,E’)** and in the BM of seminiferous tubes in male gonads **(F,F’).** Germ cells (red) were identified by the early marker OCT4 (nuclear) and late marker VASA (cytoplasmatic). The same secondary antibody was used to detect OCT4 and VASA, because the two proteins localize to different cellular compartments (nucleus and cytoplasm, respectively) and their expression can therefore be clearly distinguished. Red arrows point to the BM of the OSE, yellow arrows point to the BM of the primordial follicles, green arrows show the BM of the seminiferous tubes and ovarian cords, white arrows to the BM of blood vessels, and white asterisks marked individual germ cells expressing collagen IV. Note that occasional autofluorescent red blood cells are visible as red/orange cells inside blood vessels. Scalebars are 100 μm.

By W9-W12, the female gonad showed a higher density of germ cells in the cortex, with the occasional germ cell in the medulla and absent from the ovarian surface epithelium (OSE) (Figure 2A and 2B). Unexpectedly, already at W9, collagen IV showed a distinctive expression pattern, strongly reminiscent of the BM surrounding the developing male seminiferous tubes in gonads of similar developmental age (Figure 2C), suggesting that in females the organization of germ cells in ovarian cords occurred much earlier than previously reported [16]. Konishi and colleagues (1986) have showed by electron microscopy that germ cells were in ovarian cords in the human ovary by W15, but not at W12. Immunostaining for the ECM component collagen IV revealed that this organization in ovarian cords is in fact occurring much earlier in human development, in a process that may be common between males and females.

### Fibronectin expression in the mesenchymal compartment also revealed ovarian cords at W9-W12

**Figure 3:**
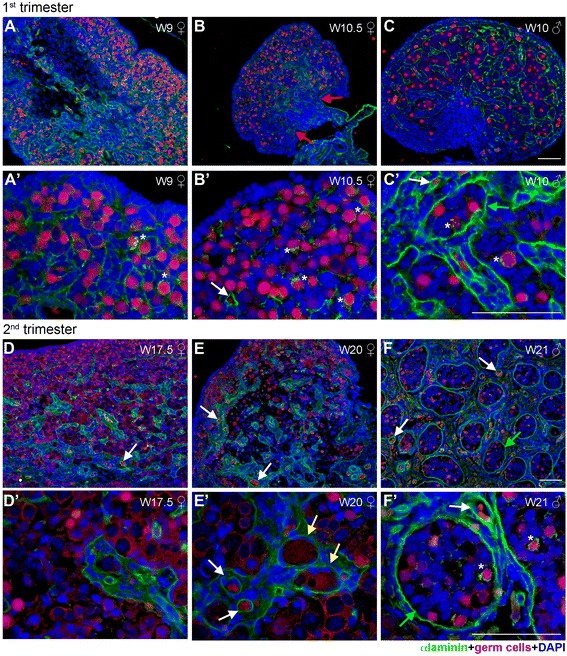
**Laminin expression in human fetal female and male gonads during first and second trimester. (A-C)** Longitudinal sight of W9 **(A, A’)** and transversal sight of W10.5 **(B, B’)** old female gonad showed a distinctive expression pattern of laminin (green). **(C, C’)** Laminin (green) detected in the BM surrounding the developing seminiferous tubes in male gonad of first trimester (W10). **(D-F)** Laminin (green) expression in the BM of ovarian cords in female gonads **(D, D’, E, E’)** and in the BM of seminiferous tubes in male gonads **(F, F’).** Germ cells (red) were identified by the early marker OCT4 (nuclear) and late marker VASA (cytoplasmatic). The same secondary antibody was used to detect OCT4 and VASA, because the two proteins localize to different cellular compartments (nucleus and cytoplasm, respectively) and their expression can therefore be clearly distinguished. Red arrows point to the BM of the OSE, yellow arrows point to the BM of the primordial follicles, green arrows show the BM of the seminiferous tubes**,** and white arrows to the BM of blood vessels, and white asterisks marked individual germ cells expressing laminin. Note that occasional autofluorescent red blood cells are visible as red/orange cells inside blood vessels. Scalebars are 100 μm.

**Figure 4:**
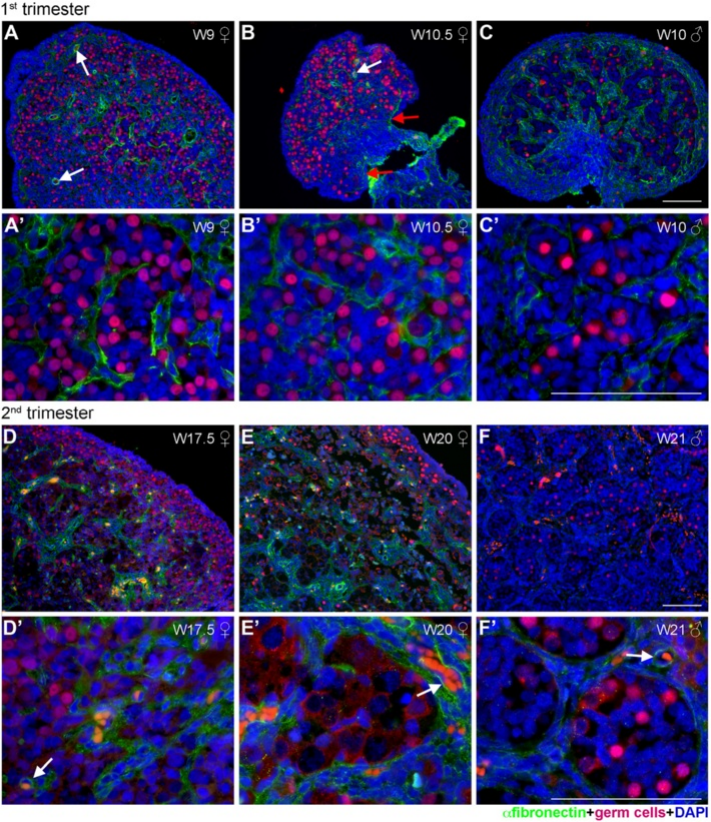
**Fibronectin expression in human fetal female and male gonads during first and second trimester. (A-F)** Fibronectin (green) expression in the mesenchymal compartment of developing female gonads **(A, A’, B, B’, D, D’, E, E’)** and male gonads **(C, C’, F, F’)** during first and second trimester. Germ cells (red) were identified by the early marker OCT4 (nuclear) and late marker VASA (cytoplasmic). The same secondary antibody was used to detect OCT4 and VASA, because the two proteins localize to different cellular compartments (nucleus and cytoplasm, respectively) and their expression can therefore be clearly distinguished. Red arrows point to the BM of the OSE, and white arrows point to the BM of blood vessels. Note that occasional autofluorescent red blood cells are visible as red/orange cells inside blood vessels. Scalebars are 100 μm.

**Figure 5:**
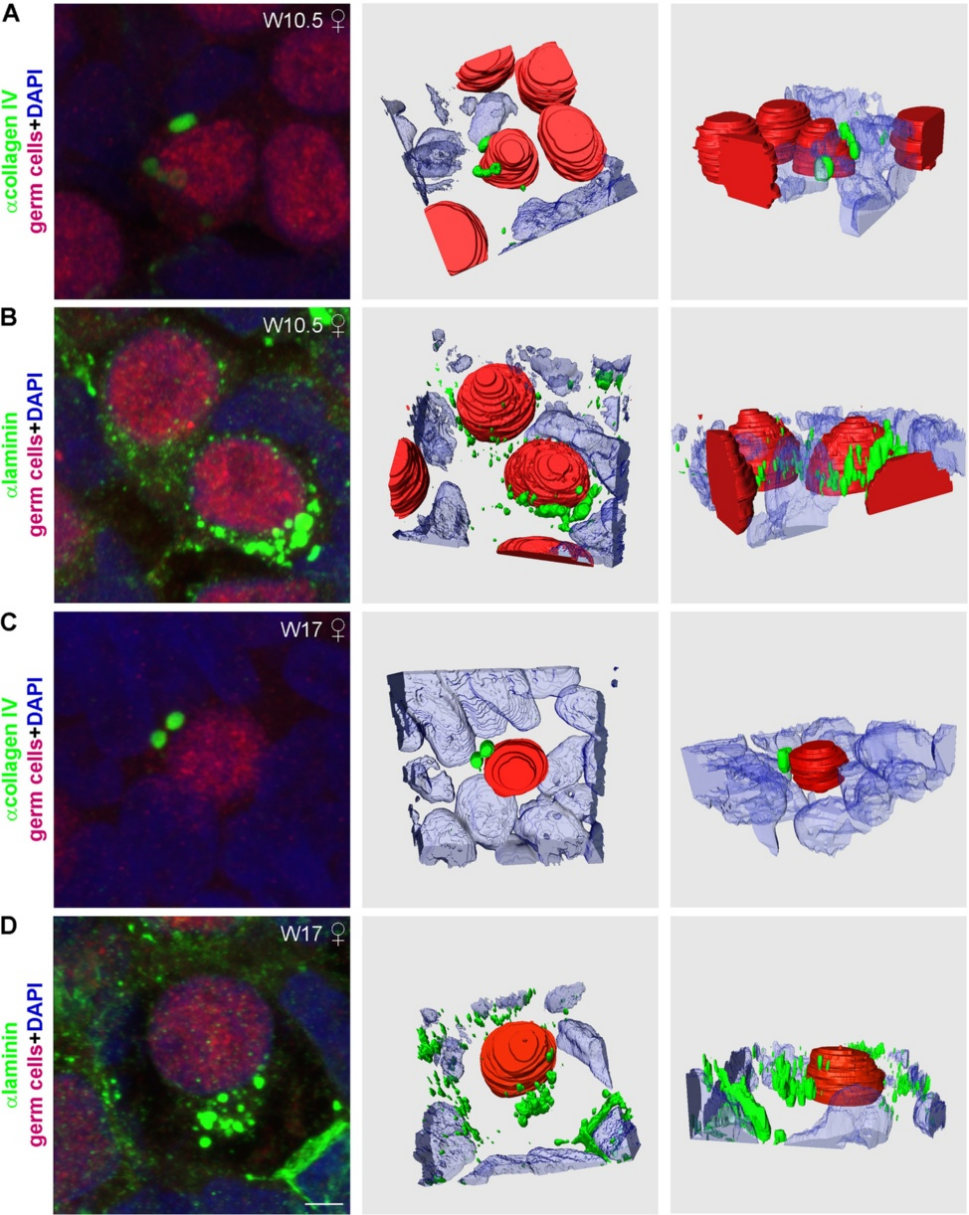
**Collagen IV and laminin granules in human fetal germ cells.** Collagen IV **(A,C)** and laminin **(B,D)** granules (in green) in the cytoplasm of germ cells (red) in both developing female first trimester (W10.5) and second trimester (W17) gonads. The same secondary antibody was used to detect OCT4 and VASA, both germ cell markers, because the two proteins localize to different cellular compartments (nucleus and cytoplasm, respectively) and their expression can therefore be clearly distinguished. 3D reconstructions from high magnification confocal z-projections from two different angles show that these granules are present throughout the cytoplasm of the germ cells. Scalebar is 2.5 μm.

To further support the existence of ovarian cords at W9-W12, we have immunostained female gonads for the two other main components of ECM, laminin (Figure 3) and fibronectin (Figure 4). Laminin was highly expressed throughout the cortex of the ovary with no specific enrichment in ovarian BM, except for the BM enveloping the developing blood vessels (white arrow in Figure 3B’); whereas in males, laminin was concentrated in the BM of the seminiferous cords and the blood vessels (green and white arrows in Figure 3C’). Moreover, we noted that few scattered (male and female) germ cells seemed to contain prominent collagen IV-rich and laminin-rich granules in their cytoplasm (white asterisks in Figures 1B, 1C, 2A’, 2B’, 3A’, 3B’, and 3C’). By performing 3D reconstructions on confocal images, we confirmed that the granules were indeed in the cytoplasm of female germ cells (Figure 5A and 5B) and male germ cells (data not shown). In general, the granules of laminin seemed more numerous than those of collagen IV.

In W9-W12 females, similarly to laminin, fibronectin marked the BM of the developing blood vessels (white arrows in Figure 4A and 4B). However, in contrast to the ubiquitously expression of laminin, fibronectin was diffusely expressed but exclusively by the mesenchymal compartment of the ovarian cortex, revealing the structure of fibronectin-negative ovarian cords (Figure 4A and 4B). This expression pattern was similar to that observed in males (Figure 4C), suggesting that the deposition of ECM created a clear physical separation between the (cortical) mesenchymal compartment (containing fibroblasts and blood vessels) and the ovarian cords in females and seminiferous tubes in males in a similar fashion in both sexes around W9-W12.

### No ECM-rich basement membrane separating the OSE from the cortex

At W9-W12, we expected to observe of a clear BM between the OSE and the cortex. However, this was only the case in a small area of the OSE close to the region of the ovarian hilus, showing a BM of collagen IV, laminin and fibronectin (red arrows in Figures 2B, 3B, and 4B), but not in the region of the OSE surrounding the ovarian cortex (Figures 1, 2, and 3). This suggested that due to the absence of a BM the cells of the OSE may still be able to traffic easily into the cortex.

### At W17-W20, male and female gonads still show similarities in organization

At W17-W20, we observed two distinct regions in the ovarian cortex (zone 1 and zone 2) (Figure 2D and 2E). This was previously observed by Anderson and colleagues [17] that showed that in the second trimester ovaries peripheral germ cells were exclusively marked by the early markers OCT4 or NANOG (zone 1), whereas more interiorly located germ cells were exclusively marked by the late markers VASA or DAZL (zone 2), with very few germ cells showing colocalization. As we were interested in the total population of germ cells in the gonad, we used both early and late germ cell markers (OCT4 + VASA) combined, making use of the fact that OCT4 and VASA have different cellular localizations, nuclear and cytoplasmatic respectively.

The region of the cortex closer to the surface (zone 1) was morphological similar to that of the first trimester cortex (Figure 2D and 2E). However, in the more interior region of the cortex (zone 2), the separation between the mesenchymal and the ovarian cords became now even more pronounced by collagen IV and laminin (Figures 2D, 2E, 3D and 3E). In zone 2, the ovarian cords increased in diameter, as the germ cells matured and some, by their nucleus morphology, seemed to have entered meiosis; whereas the mesenchymal compartment of the cortex also increased in size and contained numerous blood vessels surrounded by a collagen IV-rich and laminin-rich BM (white arrows in Figures 2D, 2D’, 2E, 2E’, 2F, 2F’, 3D, 3E, 3E’, 3F, 3F’ and Additional file 1: Figure S1). Interestingly, some of the ovarian cords still closely resembled the organization of the seminiferous tubes (Figures 2D, 2E, 2F, 3D, 3E and 3F), although the ovarian cords seemed to contain a relatively higher density of germ cells. Moreover, the expression of collagen IV and laminin showed that the female mesenchymal compartment of the cortex was more compacted and seemed more irrigated with blood vessels than in the males of similar developmental age (Figures 2D, 2E, 2F, 3D, 3E, 3F and Additional file 1: Figure S1). Furthermore, collagen IV and laminin-rich granules were also present in the cytoplasm of second trimester female germ cells (Figure 5C and 5D) and male germ cells (3F’, data for collagen IV not shown). Both in males and females, collagen IV concentrated in BMs, whereas laminin was present in BMs and expressed diffusely in the mesenchymal compartment (Figures 2D, 2E, 2F, 3D, 3E and 3F). Using available Deep-Sequencing data from FACS-sorted male and female germ cells from W18-W18.5 (16–16.5 weeks of development) [18], we confirm that germ cells express high levels of many chains of laminin and collagen IV (Additional file 2: Figure S2A). This in contrast to adult mature oocytes, that express laminin B1 and fibronectin, but particularly high levels of ZP3 [19] (Additional file 2: Figure S2B).

As in the gonads in the first trimester, at W17-W20, fibronectin was also diffusely expressed by the cells of the mesenchymal compartment in females (Figure 4D and 4E) and was detected in the BM of developing blood vessels (white arrow in Figure 4D’ and 4E’). This pattern was similar to that observed in the male gonads, but the expression of fibronectin in males was generally very faint (Figure 4F and 4F’).

### Follicular BM developed in the fetal female gonads at W17-W20

One major structural difference between gametogenesis in males and females is the development of individually encapsulated primordial follicles in females, consisting of a single germ cell (or oocyte) surrounded by one layer of squamous granulosa cells. Around W17-W20, we observed emerging primordial follicles for the first time at a characteristic location, individually embedded in the (cortical) mesenchymal compartment, physically separated from the rest of the germ cells in the ovarian cords. Moreover, those emerging primordial follicles were clearly surrounded by a BM of collagen IV (yellow arrows in Figure 2E’). Therefore, the BM delimited-(cortical) mesenchyme compartment contained BM-delimited blood vessels and BM-delimited primordial follicles. The BM surrounding the primordial follicles in the (cortical) mesenchymal compartment were also laminin-positive (yellow arrows in Figure 3E’).

Together, collagen IV, laminin and fibronectin demarked the development of the (cortical) mesenchymal compartment in the female gonads during the first and, even more pronounced, during the second trimester facilitating the embedding of blood vessels and more importantly the assembly of primordial follicles.

### Collagen IV, laminin and fibronectin expression in adult ovary

**Figure 6:**
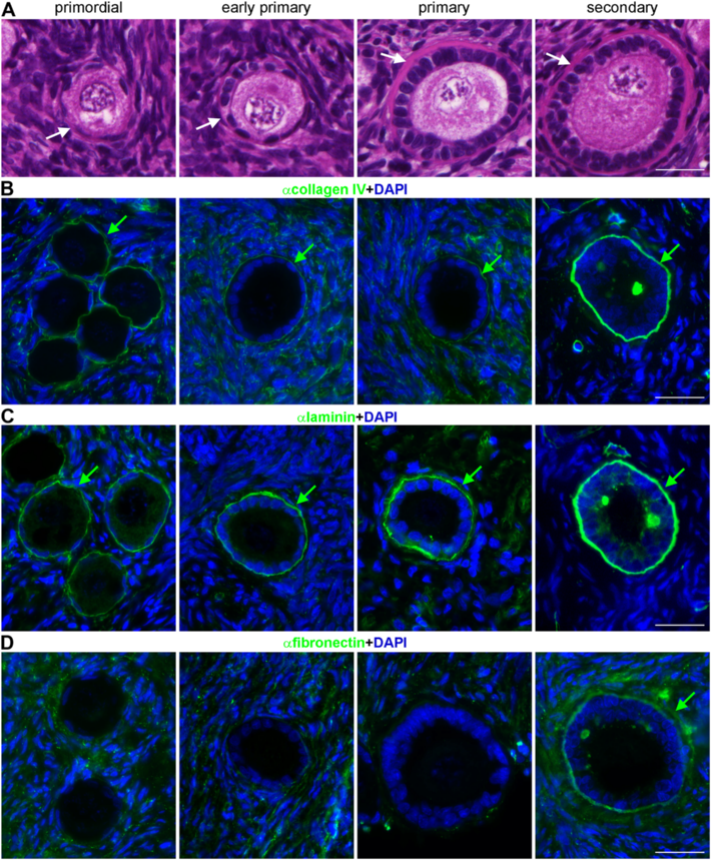
**Composition of the BM in early stage follicles in human ovarian tissue. (A)** Histological analysis of human ovarian tissue revealed an extracellular BM (white arrows) surrounding primordial, early primary, primary and secondary follicles. **(B)** Collagen IV (green) expression in the BM (green arrows) of primordial, early primary, primary and in secondary follicles. **(C)** Laminin (green) expression in the BM (green arrows) of primordial, early primary, primary and in secondary follicles. **(D)** Fibronectin (green) expression in the mesenchymal compartment of the ovarian cortex. Sporadically, we observed fibronectin expression in the BM of secondary follicles (green arrow). Scalebars are 25 μm.

We next investigated the presence of collagen IV, laminin and fibronectin in human adult ovaries during early folliculogenesis. After Haematoxylin and Eosin (H&E) staining, an increasingly thick BM was observed surrounding the developing follicles, from primordial, to early primary, to primary and finally secondary follicles (Figure 6A). This BM was collagen IV-rich (Figure 6B) and laminin-rich (Figure 6C).

**Figure 7:**
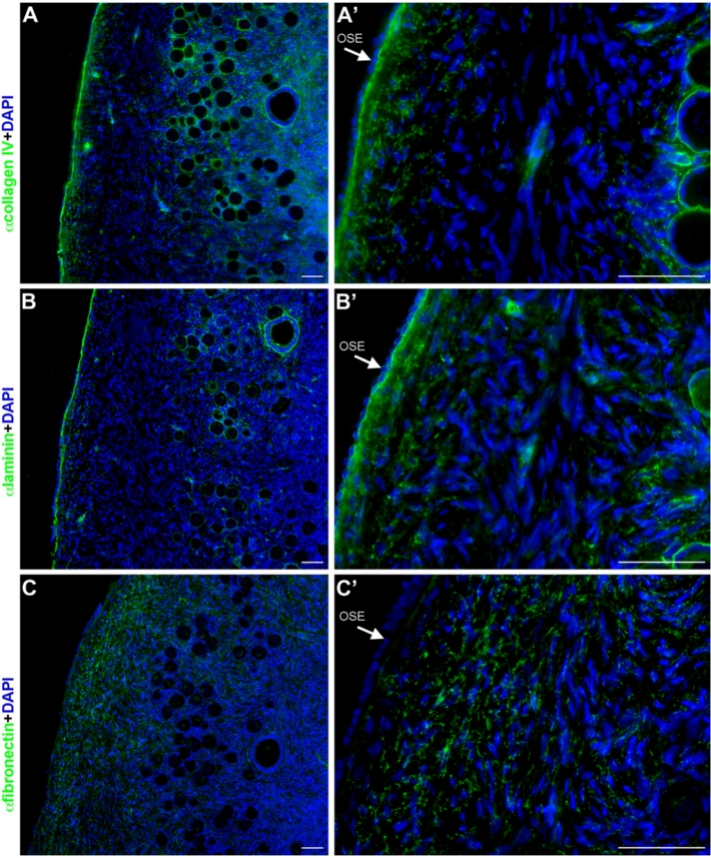
**Extracellular matrix components in the cortex of human adult ovarian tissue. (A-C)** Collagen IV **(A, A’)**, laminin **(B, B’)** and fibronectin **(C, C’)** expression (in green) in the BM underlying the OSE (white arrows) in the adult ovarian cortex. Scalebars are 50 μm.

As in the fetal gonads, fibronectin expression was diffusely observed throughout the (cortical) mesenchyme and sporadically in the BM of secondary follicles (Figure 6D). Collagen IV, laminin and fibronectin expression was also detected diffusely throughout the (cortical) mesenchymal compartment of the ovarian cortex, but in particular in the tunica albuginea (Figure 7). Moreover, collagen IV and laminin, but not fibronectin, showed prominent expression in the BM under the OSE (Figure 7).

## Discussion

### ECM revealed a higher degree of organization in human developing gonads

**Figure 8:**
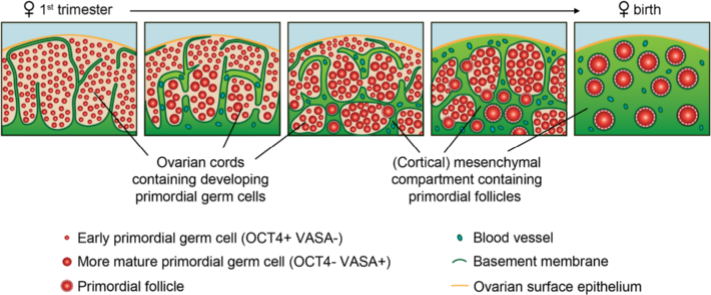
**Model of ovarian cords and mesenchymal compartment organization in fetal ovaries during gonadogenesis up to early folliculogenesis.** In female gonads, a specific organization in ovarian cords appear. The majority of germ cells in the first trimester ovary are early germ cells, but in the second trimester both early and more mature germ cells (morphological bigger germ cells) were present and segregated spatially in the ovary: early germ cells located closer to the surface whereas the more mature (bigger) germ cells are located in the inner part of the cortex [17]. From W17, individual primordial follicles become embedded in the mesenchymal compartment and at birth all germ cells are present as individual primordial follicles in the mesenchymal compartment which supports the further development of these follicles up to ovulation in adulthood.

Here, we show that basically from the time the PGCs arrive to the gonads (in both male and females), the ECM contributed to create a “germ cell” niche where the germ cells could develop their sex-specific characteristics (Figure 8). More importantly, not only in males, but also in females a specific organization in germ cell cords became apparent after immunostaining for ECM components at W10. Our results in human are in agreement with a recent study in cow [20], suggesting a higher degree of organization in female gonads and much earlier than previously thought [16]. Well-defined ovarian cords have also been described in other mammalian species, like pig and sheep [1,21].

Interestingly, a strong ECM layer between the surface epithelium of the developing ovary and the cortex was not observed, whereas this was clearly the case with the surface epithelium contacting the hilus of the developing ovary. This is in agreement with studies proposing that cells from the surface of the developing ovary could be a common precursor for both the somatic cells within the ovarian cords (becoming granulosa cells) and the cells of the adult OSE [20,22,23].

### Primordial follicles contain a collagen IV-rich and laminin-rich basement membrane

In line with the suggestion that ECM contributes to the generation of a microenvironment important for follicle integrity, we report here that in humans, as in cow [20], the primordial follicles became individually embedded in the ECM-rich (cortical) mesenchymal compartment as they were formed (Figure 8). It is interesting to note that both the capillaries and primordial follicles present in the (cortical) mesenchymal compartment showed a strong collagen IV-rich and laminin-rich BM.

Multiple collagen IV-rich granules and laminin-rich granules were observed in the cytoplasm of germ cells in the first and second trimester in both females and males. It has been proposed that ectoderm-derived cells are able to produce collagens, laminins and glycosaminoglycans, whereas fibronectin is produced mainly by mesenchymal cells [24]. Our study suggests that human germ cells, in both female and male gonads, may be able to produce both collagen IV-rich granules and laminin-rich granules perhaps directly contributing to the formation of the prominent basement membranes delineating the seminiferous tubules, the ovarian cords and (at least primordial) follicles.

### ECM composition during (early) folliculogenesis in humans and other mammals

In human, we observed collagen IV staining in primordial to primary to secondary follicles, which is consistent with studies in cow and mouse [25-27], but contrasts with a study in mouse where collagen IV was not observed in the BM of early follicles [9]. During late folliculogenesis, collagen IV, albeit with different levels of expression for the different chains, has been described in the BM of antral follicles in cow [25], mouse [9,26] and human [13,14].

The pattern of fibronectin expression in humans suggested that it may play an important role in defining the (cortical) mesenchymal compartment in particular during fetal stages, but it has a much less prominent role in the formation of the follicular basement membranes in human, mouse [9] and cow [20].

## Conclusions

Our study reveals that the human ovary is compartmentalized earlier in development than previously thought and that it resembles the male gonads regarding the distribution of the main ECM components. In addition to data showing a spatial progression in the development of germ cells starting from the inner to the outer part of the ovarian cortex [17], we now show that the formation or assembly of primordial follicles occurs in the (cortical) mesenchymal compartment (Figure 8) accompanied by the formation of a follicular BM. The BM becomes more prominent as the follicles develop during adulthood. We provide an initial characterization of the molecular niche during human gametogenesis and early folliculogenesis *in vivo* and this may prove important to develop successful protocols to progress through folliculogenesis *in vitro*. We envisage that *in vitro* maturation could occur more efficiently in 3D scaffolds integrating ECM components to facilitate and support the integrity and growth of the follicles and their basement membranes.

## Methods

### Ethical approval for human ovarian tissue and fetal gonads

The human adult ovary tissue pieces used in this study (n = 10) were obtained from cancer patients of reproductive age (Additional file 3: Table S1) that underwent unilateral oophorectomy. The use of human tissues was approved by the Medical Ethical Committee of the Leiden University Medical Center (CME 05/03 K/YR) and patients have signed an informed consent. All human embryos used in this study were donated for research with informed consent. Human fetal testes (W8.3-W21, n = 16) and human fetal ovaries (W6.6-W21.5, n = 15) were collected from volunteer abortions without medical indications (Additional file 3: Table S1). Both collection and use of human fetal gonads was approved by the Medical Ethical Committee of the Leiden University Medical Center (P08.087).

### Histology and sex identification

Fetal gonads were fixed in 4% paraformaldehyde (PFA) (MERCK, Darmstadt, Germany) overnight (o/n) at 4°C and adult ovarian pieces were fixed either in Bouin’s solution (Sigma-Aldrich, St. Louis, USA) or 4% PFA o/n at 4°C. Thereafter, the tissues were washed 3× in phosphate-buffered saline (PBS), transferred to 70% ethanol solution and embedded in paraffin using a Shandon Excelsior tissue processor (Thermo Scientific, Altrincham, UK). The material was sectioned (5 μm) using a RM2065 microtome (Leica Instruments GmbH, Wetzlar, Germany) and mounted using Prolong Gold (Life Technologies, Carlsbad, USA) on StarFrost slides (Waldemar Knittel, Braunschweig, Germany). The sex of the human fetal gonads was determined using a genomic PCR for AMELOGENIN (FW 5′ CTG ATG GTT GGC CTC AAG CCT GTG 3′ and RV 5′-TAA AGA GAT TCA TTA ACT TGA CTG-3′), that resulted in two different sized amplicons when from the X (977 bp) or Y (790 bp) chromosomes [28]. The PCR was performed using SilverStar Taq DNA polymerase (Eurogentec, Seraing, Belgium) with a PCR cycle of 95°C for 5 minutes, 34 times 95°C for 1 minutes, 60°C for 30 seconds, 72°C for 2 minutes and a final extension step at 72°C for 10 minutes. The PCR products were run on a 1.5% agarose gel.

### Histochemistry and immunofluorescence

The paraffin sections were deparaffinised in xylene and hydrated in series of decreasing concentrations of ethanol (2× 100%, 2× 90%, 80% and 70%) followed by milli-Q water. For H&E, the slides were stained in Haematoxylin (MERCK, Darmstadt, Germany) for 3 minutes at RT, washed 10 minutes with running tap water, and stained with Eosin (MERCK, Darmstadt, Germany) for 1 minute at RT. The slides were dehydrated in 2× 100% ethanol, followed by 3× 5 minutes in xylene, and mounted with Entellan (MERCK, Darmstadt, Germany). The H&E stained slides were scanned in totality on a Panoramic MIDI digital scanner (3DHISTECH Ltd., Budapest, Hungary) and representative areas were selected for images using the software program ‘Panoramic viewer’ (3D HISTECH, Budapest, Hungary).

For antigen retrieval, deparaffinised sections were either heated in 0.01 M citric buffer pH6.0 on a Tissuewave 2 microwave (Thermo Scientific, Altrincham, UK) at 98°C for 12 minutes and allowed to cool down for 30 minutes at RT; or treated with 20 μg/ml proteinase K (Promega, USA) in TE-CaCl_2_ buffer pH8.0 (50 mM Tris Base, 1 mM EDTA, 5 mM CaCl_2_, 0.5% Triton-X) for 5–10 minutes at RT. Thereafter, the slides were washed 2× in PBS and incubated with blocking buffer consisting of 1% bovine serum albumin (BSA) (Life Technologies, Carlsbad, USA) in 0.05% Tween (MERCK, Darmstadt, Germany) in PBS for 1 hour at RT. Subsequently, the sections were incubated with primary antibodies diluted in blocking buffer o/n at 4°C. The primary antibodies used were goat anti-VASA (1:1000, AF2030, R&D systems, Minneapolis, USA); goat anti-OCT4 (1:100, sc-8628, Santa Cruz Biotechnology, Dallas, USA); rabbit anti-collagen IV (1:50, AB748, Millipore, Billerica, USA); rabbit anti-laminin (1:50, Z0097, DakoCytomation, Glostrup, Denmark), rabbit anti-fibronectin (1:400, F3648, Sigma-Aldrich, St. Louis, USA) and mouse anti-CD31 (1:100, M0823, Dako, Glostrup, Denmark). The next day, the slides were washed twice in PBS, once in 0.05% Tween/PBS and incubated with Alexa Fluor 594 donkey anti-goat (1:500, A11058, Life Technologies, Carlsbad, USA), Alexa Fluor 594 donkey anti-mouse (1:500, A21203, Life Technologies, Carlsbad, USA) and Alexa Fluor 488 donkey anti-rabbit (1:500, A21206, Life Technologies, Carlsbad, USA) diluted in blocking buffer for 2 hours at RT. Finally, the slides were washed twice in PBS, counterstained with 4′,6-diamidino-2-phenylindole dihydrochloride (DAPI) (Life Technologies, Carlsbad, USA) for 1 minute and mounted under coverslips with ProLongGold antifade reagent (Life Technologies, Carlsbad, USA). Negative controls were performed by omitting the primary antibodies (Additional file 4: Figure S3).

### Imaging and 3D reconstructions

Slides were analysed on a Leica DMRA fluorescence microscope (Leica, Wetzlar, Germany) and pictures were made with the CoolSnap HQ2 camera (Photometrics, Tucson, USA). For high magnifications and 3D reconstructions, slides were scanned with Leica SP8 upright confocal microscope (Leica) using the Leica Application Suite Advanced Fluorescence software (LAS AF, Leica) using a Leica objective 100×/1.4 oil HCX PL Fluotar and 6× digital zoom. Z-steps with sample density according to the Nyquist rate were performed and Z-projections were generated. Amira software (version 4.1; Visage Imaging, San Diego, USA) was used for 3D reconstruction. Figures were compiled using Photoshop CS5 (Adobe Systems Inc., San Jose, USA).

### Gene expression data

Expression data of genes of interest of FACS-sorted (cKIT-positive) germ cells from W18-W18.5 (16–16.5 weeks of development) testis and ovary as well as (TRA-1-60-positive) H1 human embryonic stem cells were obtained from GSE39821 [18]. To evaluate the expression levels, the count data (counts per million (CPM)) was analysed with the edgeR package (version 3.8.8) [29,30] in R (version 3.1.1). The reads per kilobase per million (RPKM) of genes of interest from human adult mature oocytes were obtained from the supplementary information published by Yan and colleagues [19]. Data were visualized using the R package ggplot2 (version 1.0.0) [31].

## Additional files

Additional file 1: Figure S1. A BM of collagen IV and laminin mark the (CD31-positive) vasculature in human gonads. CD31-positive blood vessels present in the mesenchymal compartment are marked by a BM of collagen IV (in green) (A,B) and laminin (in green) (C,D) in both female (W20) and male (W21) gonads from second trimester. White arrows point to the BM of blood vessels. Note that occasional autofluorescent red blood cells are visible as red/orange cells inside blood vessels. Scalebar is 10 μm. Additional file 2: Figure S2. Gene expression levels of ECM components, germ cell markers and housekeeping genes in second trimester germ cells and mature oocytes. (A) Gene expression levels of ECM components (laminin chains, collagen IV chains, fibronectin (FN)), zona pellucida glycoprotein 3 (ZP3) (left and middle panel), germ cell markers (PRDM14, KIT (c-Kit), DDX4 (VASA), DAZL, and SYCP3), and housekeeping genes (ACTB, GAPDH, and HSP90AB1) (right panel) in cKIT-positive (cKIT+) germ cells from W18-W18.5 (16–16.5 weeks of development) testis and ovary (two replicates each) and TRA-1-60-positive (TRA-1-60+) H1 human embryonic stem cells (in triplicate) [18]. Gene expression levels are represented as counts per million (CPM). (B) Gene expression levels of the same genes as in (A) in three human adult mature oocytes [19]. Gene expression levels are represented as reads per kilobase per million (RPKM). Additional file 3: Table S1. Characteristics of the collected material. Additional file 4: Figure S3. Collagen IV expression and negative controls of fetal gonads and adult ovary. Negative controls were performed on the fetal gonads and adult ovarian tissue pieces used here (Additional file 3: Table S1) by omitting the primary antibodies. Depicted are some examples of female and male gonads from the first and second trimesters as well as adult ovarian tissue immunostained for collagen IV (green) in combination with VASA/OCT4 (red) on the left and the respective negative control on the right. Scalebar is 100 μm.

## Abbreviations

BM: Basement membrane
ECM: Extracellular matrix
OSE: Ovarian surface epithelium
PGCs: Primordial germ cells
H&E: Haematoxylin and eosin
