# Supplementary figures and images for: Development of the follicular basement membrane during human gametogenesis and early folliculogenesis
Source: BMC Dev Biol. 2015 Jan 21;15:4. doi: 10.1186/s12861-015-0054-0 (PMC4307144; doi:10.1186/s12861-015-0054-0)

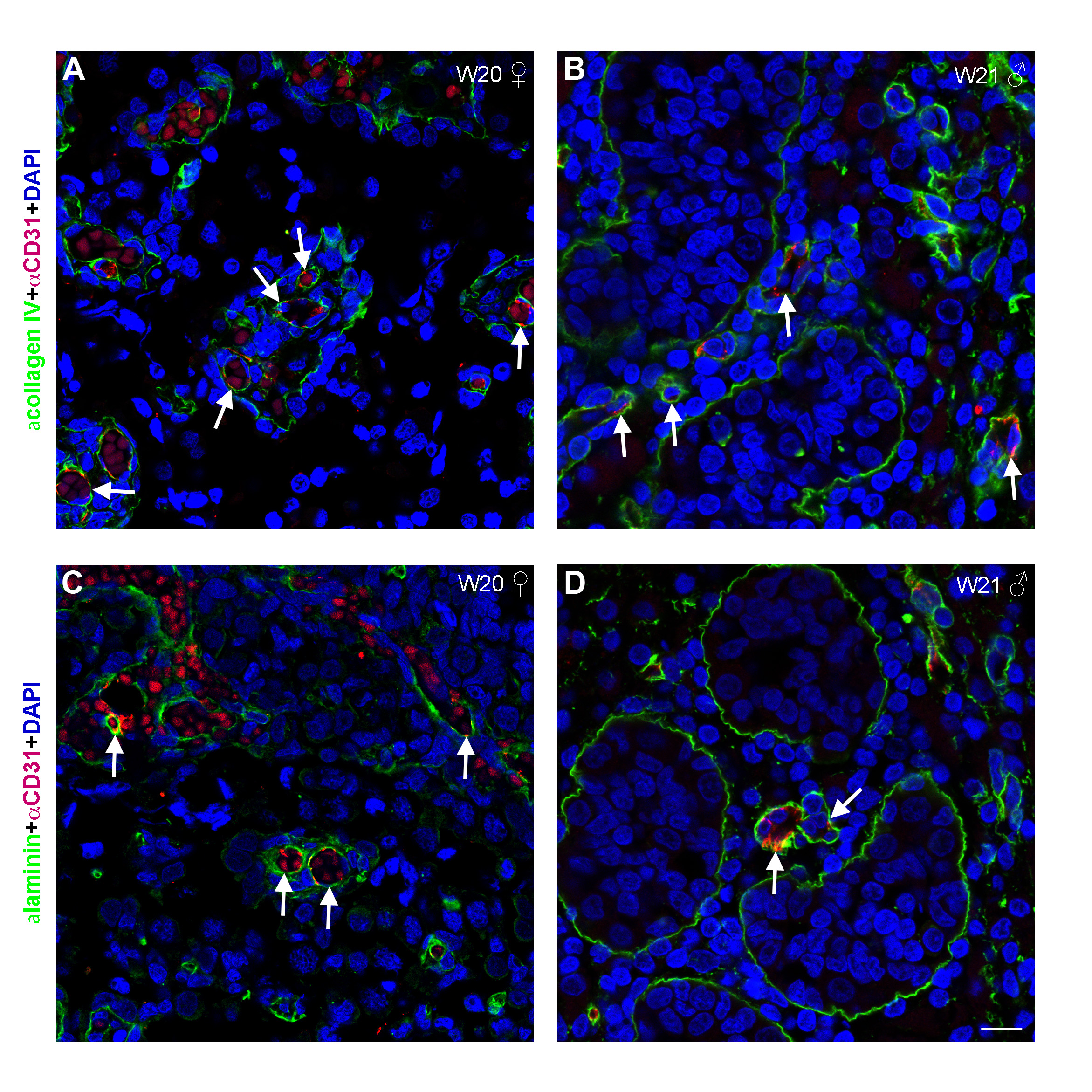

Supplement: Additional file 1: Figure S1. — A BM of collagen IV and laminin mark the (CD31-positive) vasculature in human gonads. CD31-positive blood vessels present in the mesenchymal compartment are marked by a BM of collagen IV (in green) (A,B) and laminin (in green) (C,D) in both female (W20) and male (W21) gonads from second trimester. White arrows point to the BM of blood vessels. Note that occasional autofluorescent red blood cells are visible as red/orange cells inside blood vessels. Scalebar is 10 μm. [file 12861_2015_54_MOESM1_ESM.jpeg]

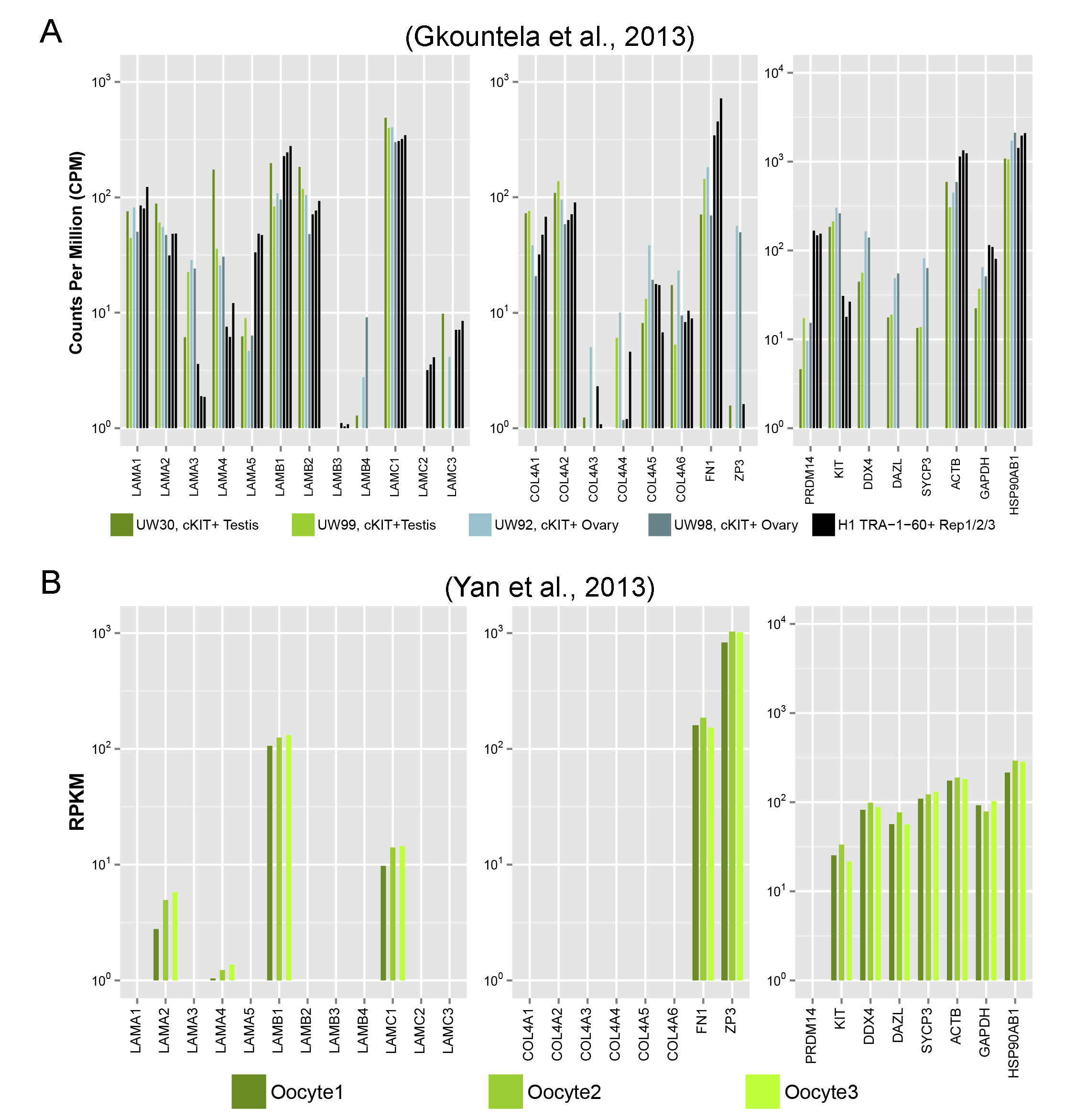

Supplement: Additional file 2: Figure S2. — Gene expression levels of ECM components, germ cell markers and housekeeping genes in second trimester germ cells and mature oocytes. (A) Gene expression levels of ECM components (laminin chains, collagen IV chains, fibronectin (FN)), zona pellucida glycoprotein 3 (ZP3) (left and middle panel), germ cell markers (PRDM14, KIT (c-Kit), DDX4 (VASA), DAZL, and SYCP3), and housekeeping genes (ACTB, GAPDH, and HSP90AB1) (right panel) in cKIT-positive (cKIT+) germ cells from W18-W18.5 (16–16.5 weeks of development) testis and ovary (two replicates each) and TRA-1-60-positive (TRA-1-60+) H1 human embryonic stem cells (in triplicate) [18]. Gene expression levels are represented as counts per million (CPM). (B) Gene expression levels of the same genes as in (A) in three human adult mature oocytes [19]. Gene expression levels are represented as reads per kilobase per million (RPKM). [file 12861_2015_54_MOESM2_ESM.jpeg]

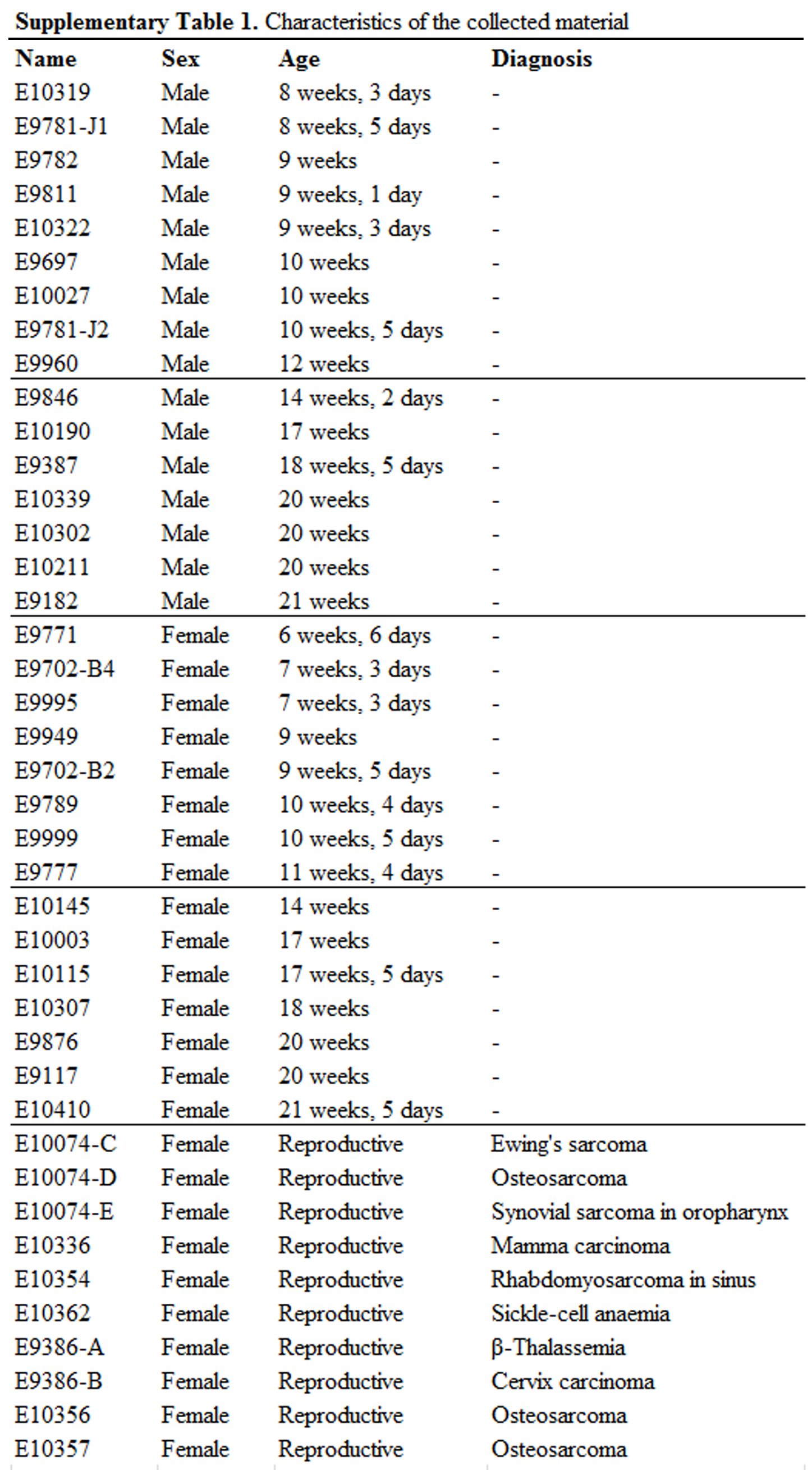

Supplement: Additional file 3: Table S1. — Characteristics of the collected material. [file 12861_2015_54_MOESM3_ESM.jpeg]

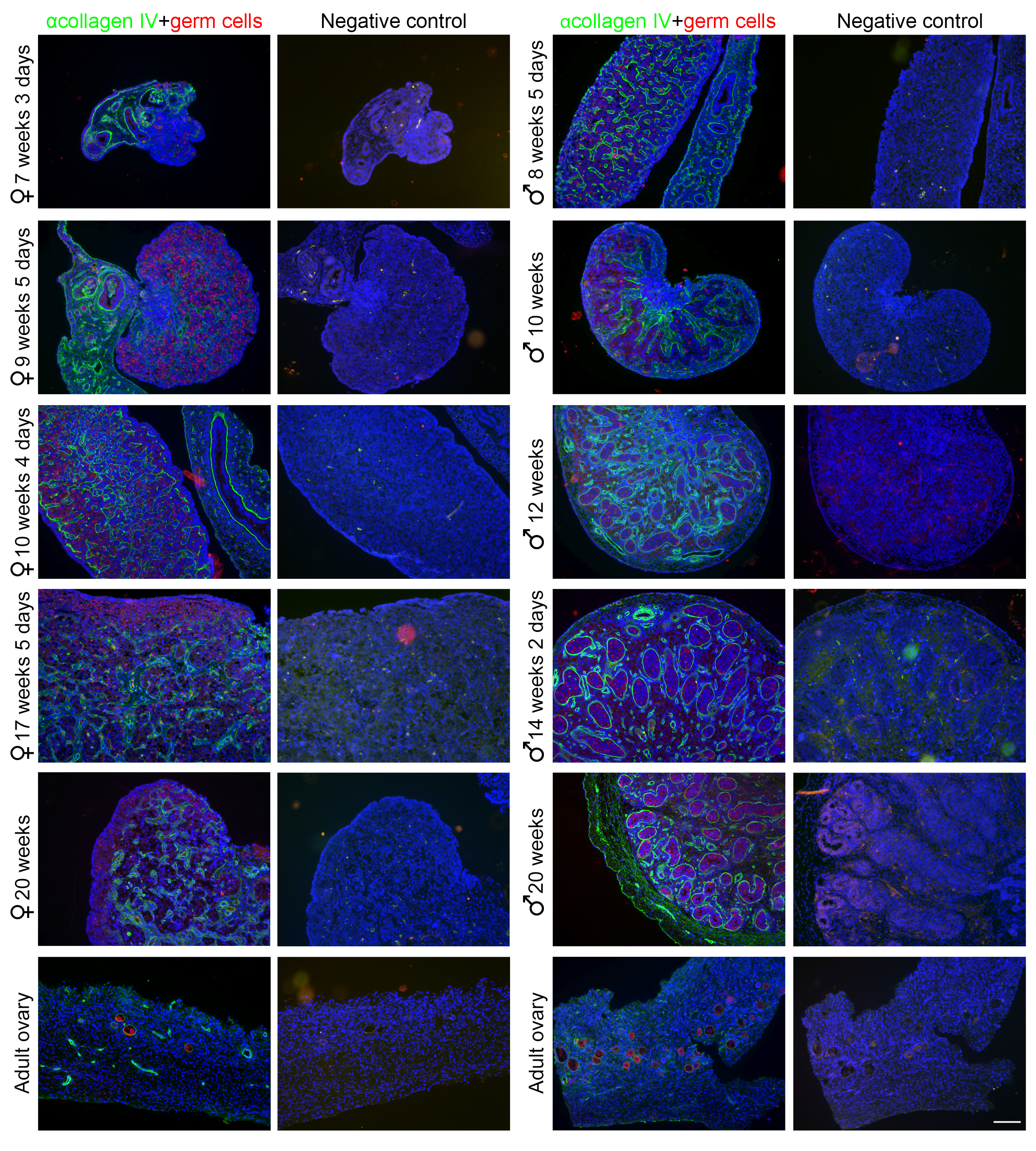

Supplement: Additional file 4: Figure S3. — Collagen IV expression and negative controls of fetal gonads and adult ovary. Negative controls were performed on the fetal gonads and adult ovarian tissue pieces used here (Additional file 3: Table S1) by omitting the primary antibodies. Depicted are some examples of female and male gonads from the first and second trimesters as well as adult ovarian tissue immunostained for collagen IV (green) in combination with VASA/OCT4 (red) on the left and the respective negative control on the right. Scalebar is 100 μm. [file 12861_2015_54_MOESM4_ESM.jpeg]
